# Strain-to-strain variation of *Rhodococcus equi* growth and biofilm formation in vitro

**DOI:** 10.1186/s13104-019-4560-1

**Published:** 2019-08-19

**Authors:** Adina R. Bujold, Nicholas R. Lani, Macarena G. Sanz

**Affiliations:** Department of Veterinary Clinical Sciences, Washington State University, Pullman, WA USA

**Keywords:** *Rhodococcus equi*, Strain-to-strain variation, Growth, Biofilm

## Abstract

**Objective:**

*Rhodococcus equi* is an opportunistic pathogen that causes disease worldwide in young foals and immunocompromised humans. The interactions of *R. equi* with the host immune system have been described; however, most studies have been conducted using a few well-characterized strains. Because biological differences between *R. equi* strains are not well characterized, it is unknown if experimental results will replicate when different strains are used. Therefore, our objective was to compare the growth and biofilm formation of low-passage-rate clinical isolates of *R. equi* to higher-passage-rate, commonly studied isolates to determine whether strain-to-strain variation exists.

**Results:**

Twelve strains were used: 103+, ATCC 33701, UKVDL206 103S harboring a GFP-expressing plasmid, a plasmid-cured 33701 (higher-passage-rate) and seven low-passage clinical isolates. Generation time in liquid revealed fast, moderate-fast, moderate-slow, and slow-growing isolates. The higher-passage-rate isolates were among the moderate-slow growing strains. A strain’s rate of growth did not correspond to its ability to form biofilm nor to its colony size on solid media. Based on our results, care should be taken not to extrapolate in vitro work that may be conducted using different *R. equi* strains. Further work is needed to evaluate the effect that the observed differences may have on experimental results.

**Electronic supplementary material:**

The online version of this article (10.1186/s13104-019-4560-1) contains supplementary material, which is available to authorized users.

## Introduction

Rhodococcal pneumonia is an important cause of morbidity and mortality in young foals worldwide and an emerging opportunistic pathogen of immunocompromised humans [1]. The etiologic agent, *Rhodococcus equi*, is a Gram-positive, aerobic, soil-dwelling opportunistic pathogen [2–4]. Inside the host, it is a facultative intracellular pathogen that replicates within macrophages, where it prevents acidification of the phagolysosome [5, 6]. Despite characterization of the immune system’s interactions with this organism and the identification of some virulence factors of *R. equi* [7], understanding of its pathogenesis is limited. The virulence-associated plasmid (VAP), and VapA in particular, is critical for virulence in horses and is routinely used to differentiate pathogenic from non-pathogenic strains [8, 9]. Virulence determinants appear to be conserved in the core genome of *R. equi* [10, 11], but differences in their expression have yet to be characterized.

Several genotypically distinct isolates of *R. equi* have been obtained from soil, air, water, and feces at horse breeding farms [12, 13], but the majority of studies to date have been conducted using a few relatively well characterized strains [2, 7, 14, 15]. The advantage to this approach is reproducibility among studies and the ability to build on previous work. However, it is hard to predict if the observed results will be replicable using different strains. Therefore, our objective was to compare the growth and biofilm formation of low-passage-rate clinical isolates of *R. equi* to commonly studied and higher-passage-rate isolates to determine whether strain-to-strain variation exists.

## Main text

### Methods

#### Bacterial strains

Strains used in this study are listed in Table 1. Strains chosen include previously studied and higher-passage-rate isolates ATCC 33701, 103+, and UKDL206 [16, 17], ATCC 33701 strain that was cured of the VAP, and a sub-cultured 103+ isolate carrying a plasmid that expresses GFP (103S-GFP). Additionally, seven low-passage clinical isolates (1–3 passages) were included. All isolates were routinely cultured from glycerol stocks stored at − 80 °C onto brain heart infusion (BHI; BD Difco, Sparks, MD) agar plates incubated at 37 °C for 24–48 h.

**Table 1:** Bacterial isolates of *Rhodococcus equi* used in this study

Isolate	Characteristics	References
103+	Isolated from pneumonic foal	[29]
103S-GFP	Subculture of 103 + strain harboring pGFPmut2 plasmid	[30]
ATCC 33701	Isolated from horse lung	[31]
ATCC 33701 pc	Plasmid cured strain of ATCC 33701	[9]
UKVDL206	Virulent clinical isolate collected by the University of Kentucky Veterinary Diagnostic Laboratory	[16]
WSU001	Clinical isolate collected from human	This study
WSU002	Clinical isolate collected from foal	This study
WSU003	Clinical isolate collected from foal	This study
WSU004	Clinical isolate collected from foal	This study
WSU005	Clinical isolate collected from foal lung	This study
WSU006	Isolated from osseus manifestation of disseminated rhodococcosis in a foal	This study
WSU007	Isolated from transtracheal wash in a foal	This study

All strains were confirmed to be *R. equi* by colony polymerase chain reaction (PCR) with crude genomic DNA isolated using Instagene matrix (Bio-Rad Laboratories Ltd., Hercules, CA), as previously described [18]. Primers targeting the *choE* gene were used to confirm the species (F: 5′-GCTCGCTTCCAGTTCAATTC-3′; R: 5′-AGCGGGTGGTATGTGAAGTC-3′; 188 bp amplicon), *vapA* primers confirmed the presence of the virulence-associated gene *vapA* (F:5′-GACTCTTCACAAGACGGT-3′ [19]; R: 5′-CGAAGTCGTCGAGCTGTCATAG-3′ [20]; 189 bp amplicon) on the virulence-associated plasmid VAP, and primers for the 16S ribosomal RNA gene were used as a global positive control (F: 5′-AGAGTTTGATCMTGGCTCAG-3′ [19]; 5′-GCTGCCTCCCGTAGGAGT-3′; 311 bp amplicon). A PCR program was run with 3 min initial denaturation at 95 °C, then 35 cycles of 30 s denaturation at 95 °C, 30 s annealing at 57 °C, and 15 s extension at 68 °C, followed by a final elongation for 6 min at 68 °C. PCR products were visualized under UV light following gel electrophoresis in a 1% agarose gel containing 1 × GelRed nucleic acid stain (Phenix Research Products, Candler, NC).

#### Growth

All isolates were grown three ways: in liquid media with shaking to measure the rate of planktonic growth; on solid media to measure colony appearance and size over time; and in static culture in 96-well microtiter plates incubated for up to 72 h, to evaluate biofilm formation.

*Growth in liquid media* For each strain, a single colony isolate from a BHI agar plate (cultured as described above) was standardized and inoculated to ~ 10^5^ colony forming units (CFU) per mL in 5 mL of pre-warmed BHI broth in a 17 × 100 mm plastic culture tube (Falcon, Corning, NY). Cultures were incubated at 37 °C with 100 rpm shaking for 40 h (until growth plateaued), OD_600nm_ was measured, and cells were enumerated by serial dilution and plate counts to determine CFU/mL immediately after inoculation (0 h post-inoculation; hpi), at 4 h intervals from 0 to 24 hpi, and again at 40 hpi. All growth curves were repeated three times for strains that grew consistently and up to six times for strains that exhibited some variation in their growth curves.

*Growth on solid media* To measure colony size development over time on solid media, BHI broth cultures for each strain were serially diluted in PBS, 100 µL was spread onto BHI agar plates and incubated at 37 °C for 48 h. Colony diameters were measured every 4 h from 24 to 48 hpi using a ruler and calipers, as previously described [21]. Experiments were repeated on three separate occasions, and each colony was treated as a biological replicate.

#### Biofilm assay

A biofilm formation assay was conducted as previously described [22], with some modifications. Briefly, cultures were inoculated as described for liquid growth curves to standardize the inoculum to an input CFU of ~ 10^5^ CFU/mL, which was confirmed by serial dilution and plate counts. For each strain, 100 µL of bacterial culture at 0 hpi were added to four replicate wells of a 96-well plate (CoStar, tissue culture-treated, Corning, NY). Two positive control strains known to form biofilms, *Staphylococcus aureus* ATCC 29213 and *Pseudomonas aeruginosa* ATCC 27853, were also added to each plate for visual comparison of biofilm formation. Four uninoculated BHI broth wells were included per plate to get a blank for calculations. For each experimental day, three identical 96-well plates were incubated at 37 °C for 24, 48, or 72 h, respectively, at which point the broth was removed by gentle suction and inversion onto paper towel. Wells were heat fixed at 56 °C for 20 min, stained with crystal violet (0.1%), washed 3–5 times with distilled water (until the water ran clear), air dried, and de-stained with 70% ethanol. Absorbance was measured at 595 nm using an Epoch plate reader (BioTek Instruments Inc., Winooski, VT), BHI wells were used to blank experimental wells and absorbance results were standardized to input CFU for each strain as previously described [22]. The assay was repeated four separate times per strain. Four empty wells per plate were also stained with crystal violet alongside experimental wells in order to confirm that the plasticware did not retain any stain.

#### Statistics

Data were analyzed using a commercial software (SigmaPlot, SPSS, Chicago, Illinois, USA). The Shapiro–Wilk test was used to test for normality. CFU/mL, colony size, and absorbance were compared using one-way analysis of variance (ANOVA) or Kruskal–Wallis one-way ANOVA on ranks. Multiple comparisons were performed using Fisher LSD or Dunn’s methods, respectively. Significance was set at p < 0.05.

All raw data collected and used for statistical analyses can be accessed in Additional file 1.

### Results and discussion

The purpose of this study was to determine whether low-passage-rate *R. equi* clinical isolates were phenotypically different than the higher-passage-rate isolates. *R. equi* is a prevalent opportunistic pathogen that causes a high financial burden on the horse industry worldwide, but there is limited information on potential differences between *R. equi* strains and the implications this may have on disease development in foals. Most studies to date have been performed using a few strains of *R. equi* (namely 103+, 33701, or UKVDL206), but here we demonstrate that there are differences between strains in the three phenotypic characteristics we evaluated. The implications these differences may have in in vitro studies remain undefined.

The low-passage-rate strains (WSU001-007) were isolated from foals except for WSU001, which was isolated from a human. All isolates were confirmed to be *R. equi* as they tested positive for *choE* by PCR. All isolates except for ATCC 33701 pc also tested positive for the *vapA* virulence-associated gene (data not shown) and are therefore considered pathogenic to horses.

**Fig. 1:**
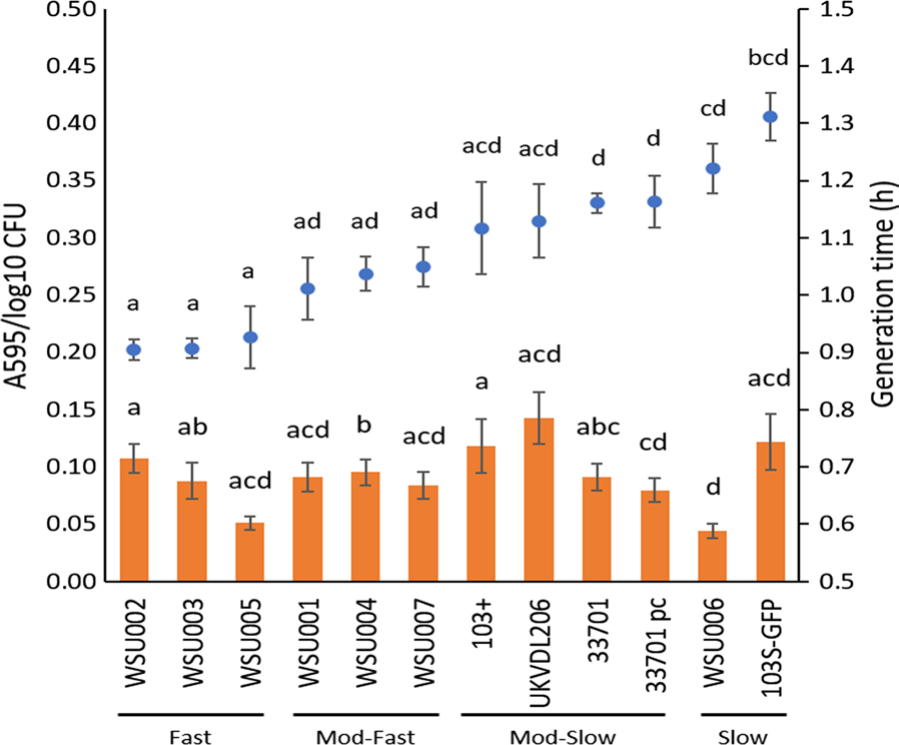
Biofilm formation of *R. equi* strains after 72 h of growth in a 96-well microtiter plate incubated at 37 °C (left axis, bars) and generation time (right axis, scatter plot). Biofilm results are normalized to log_10_ input CFU of the initial inoculum. (Error bars represent SEM. Strains with differing superscript letters are significantly different from one another, p < 0.05)

To determine whether measurable differences among strains existed, isolates were grown in liquid and on solid media, and their ability to form biofilms in vitro was measured (summarized in Table 2 and Additional file 2: Table S1). A study of *Pseudomonas aeruginosa* using the same three growth methods employed here found that biofilms more closely resemble exponentially growing planktonic cells and that solid and liquid media growth yield similar protein expression profiles [23], suggesting that these three modes of growth may provide a better in vitro model of bacterial growth than employing one method alone. Using data from liquid growth curves (Additional file 2: Fig. S1; Additional file 2: Table S2), generation times were calculated and sorted from fastest- to slowest-growing isolates (Fig. 1). Natural breakpoints in generation times divided isolates into four groups: fast (0.9–0.93 h generation times), moderate-fast (1.01–1.05 h), moderate-slow (1.12–1.16 h), or slow (1.22–1.31 h) growers. The fast and moderate-fast groups were comprised entirely of low-passage-rate isolates. The moderate-slow group contained the higher-passage-rate isolates 103+, UKVDL206, ATCC 33701, and 33701 pc. The slow group had one low-passage isolate and the sub-cultured 103S-GFP laboratory strain. The three fast strains grew significantly (p < 0.001) faster than the moderate-slow strains. The fast and moderate-fast isolates all grew significantly (p < 0.001) faster than slow isolates.

**Table 2:** Summary of results for liquid growth (generation time), solid growth (final colony diameter), and biofilm formation

Isolate	Liquid culture		Solid culture	Biofilm (A_595_/log_10_ CFU)
	Generation time (h) (fastest to slowest)		Colony diameter at 48 hpi (mm)	
WSU002	F	0.90 ± 0.02	4.56 ± 0.17	0.108 ± 0.013
WSU003	F	0.91 ± 0.02	3.50 ± 0.27	0.088 ± 0.016
WSU005	F	0.93 ± 0.05	3.95 ± 0.18	0.051 ± 0.006
WSU001	MF	1.01 ± 0.05	3.75 ± 0.15	0.091 ± 0.013
WSU004	MF	1.04 ± 0.03	5.17 ± 0.10	0.096 ± 0.011
WSU007	MF	1.05 ± 0.03	3.86 ± 0.19	0.084 ± 0.012
103+	MS	1.12 ± 0.08	3.06 ± 0.30	0.118 ± 0.024
UKVDL206	MS	1.13 ± 0.06	3.19 ± 0.23	*0.143 ± 0.023*
ATCC 33701	MS	1.16 ± 0.02	*5.38 ± 0.42*	0.091 ± 0.012
ATCC 33701 pc	MS	1.16 ± 0.04	5.08 ± 0.27	0.080 ± 0.010
WSU006	S	1.22 ± 0.04	3.30 ± 0.33	0.045 ± 0.006
103S-GFP	S	1.31 ± 0.04	1.94 ± 0.21	0.122 ± 0.024

Generation time results are shown from fastest to slowest growing strain

Italics numbers indicate the largest value among the strains

Underline indicate the smallest value among the strains

*F* fast-growing, *MF* moderate-fast-growing, *MS* moderate-slow-growing, *S* slow-growing

Differences were observed in the rate of colony appearance on solid media for the isolates studied; therefore, colony sizes were measured from 20 to 48 h to see whether this corresponded with liquid culture generation times (Table 2; Additional file 2: Fig. S2). The slow-grower 103S-GFP had the smallest colonies on solid media, but no other obvious commonalities were observed between solid and liquid growth. There have been no reports in the literature on the different rates of growth of *R. equi* strains on solid media, but colony size can be affected by the amount of capsule or extracellular polymeric substance (EPS) produced by a given strain [24]. Further examination of capsule expression would be required to validate this hypothesis.

The ability of *R. equi* to form biofilms has been previously reported [25, 26], therefore biofilm formation was examined here. Biofilm production varied greatly between strains (Fig. 1). UKVDL206 formed the most biofilm, while slow-grower WSU006 formed the least. Biofilm formation at 24 and 48 h showed no significant differences among isolates (data not shown). Using the cut-off imposed by Gressler et al. [25], whereby *R. equi* strains were deemed to be biofilm-producers if the crystal violet staining exceeded the negative control, all 12 isolates in our study were considered biofilm-producers. While this is consistent with results reported by others [27], some have reported *R. equi* strains that lack the ability to form biofilms in vitro [26]. In the present study, 103 + and 103S-GFP were relatively high biofilm formers, suggesting that the presence of the GFP plasmid did not affect this phenotype. The loss of the VAP did not affect the rate of biofilm formation of 33701, suggesting that most biofilm-associated genes are chromosomally encoded rather than plasmid-encoded. The lack of difference in growth rates between the plasmid-cured and VAP-carrying 33701 strains may be due to the fact that isolates were only grown at one temperature (37 °C). While plasmid-bearing cells have been shown to replicate slower than plasmid-cured derivatives, such differences were dependant on incubation temperature [28]. Overall, there were no obvious shared trends between growth and biofilm experiments under the conditions examined.

In summary, the 103 isolates grew more slowly in liquid, formed smaller colonies on solid media, but formed more biofilm than most strains tested. UKVDL206 grew more slowly in liquid, formed smaller-than-average colonies on solid media, but produced the most biofilm. WSU006 grew slowly and plateaued earlier than most isolates in liquid culture, produced the least biofilm, but formed slightly larger-than-average colonies on solid media. Strains 33701 and 33701 pc grew moderately-slowly but had larger colonies on solid media; however, both were only average biofilm formers. Finally, WSU007 was among the moderately-fast-growing strains but plateaued and declined early in liquid culture, had only moderately-large colonies on solid media, and was a moderate biofilm former.

### Conclusions

The results of this study demonstrate substantial strain-to-strain variation in growth rates and biofilm formation of 12 *R. equi* clinical isolates in vitro. The biological significance of our findings remains unknown; however, our study provides the fundamental basis for further research in this area. Future studies are fundamental to examining whether differences between isolates contribute to the variable outcomes of rhodococcal pneumonia and the foal’s ability to clear the infection with or without intervention.

## Limitations

- Low number of strains studied.
- Few avirulent isolates included.
- Growth in rich rather than minimal media.

## Additional files

**Additional file 1.** Raw data and calculations for the growth parameters of twelve *R. equi* isolates. Raw data collected to measure liquid and solid media growth and biofilm formation, as well as calculations done to determine the generation times in liquid media for each isolate.

**Additional file 2: Table S1.** Summary of results for liquid growth (generation time), solid growth (final colony diameter), and biofilm formation. Generation time results are shown from fastest to slowest growing strain. All other results are shown from highest to lowest value. **Table S2.** Significant differences observed between strains in liquid growth over time. **Figure S1.** Growth profiles of *R. equi* strains over time grown in BHI broth at 37 °C with 100 rpm shaking. (F: Fast-growing; MF: Moderate-fast; MS: Moderate-slow; S: Slow. Error bars represent SEM). **Figure S2.** Discrete *R. equi* colony sizes (mm) for 12 strains over time grown on BHI agar plates incubated 37ºC for 48 h. (Error bars represent SEM).

## Data Availability

All data generated or analysed during this study are included in this published article and in Additional file 1.
