# Supplementary material for: Strain-to-strain variation of Rhodococcus equi growth and biofilm formation in vitro
Source: BMC Res Notes. 2019 Aug 19;12:519. doi: 10.1186/s13104-019-4560-1 (PMC6701102; doi:10.1186/s13104-019-4560-1)
Supplement: Supplementary file 2 — Additional file 2: Table S1. Summary of results for liquid growth (generation time), solid growth (final colony diameter), and biofilm formation. Generation time results are shown from fastest to slowest growing strain. All other results are shown from highest to lowest value. Table S2. Significant differences observed between strains in liquid growth over time. Figure S1. Growth profiles of R. equi strains over time grown in BHI broth at 37 °C with 100 rpm shaking. (F: Fast-growing; MF: Moderate-fast; MS: Moderate-slow; S: Slow. Error bars represent SEM). Figure S2. Discrete R. equi colony sizes (mm) for 12 strains over time grown on BHI agar plates incubated 37ºC for 48 h. (Error bars represent SEM). [file 13104_2019_4560_MOESM2_ESM.docx]

**Additional Figures and Tables**

**Table S1.** Summary of results for liquid growth (generation time), solid growth (final colony diameter), and biofilm formation. Generation time results are shown from fastest to slowest growing strain. All other results are shown from highest to lowest value.

| **Isolate** | **Liquid culture** | | **Solid culture** | **Biofilm** |
| --- | --- | --- | --- | --- |
|  | **Generation time**  **(fastest to slowest)** | | **Colony diameter at 48 hpi** |  |
| WSU002 | **F** | WSU002 | ATCC 33701 | UKVDL206 |
| WSU003 | **F** | WSU003 | WSU004 | 103S-GFP |
| WSU005 | **F** | WSU005 | ATCC 33701 pc | 103+ |
| WSU001 | **MF** | WSU001 | WSU002 | WSU002 |
| WSU004 | **MF** | WSU004 | WSU005 | WSU004 |
| WSU007 | **MF** | WSU007 | WSU007 | WSU001 |
| 103+ | **MS** | 103+ | WSU001 | ATCC 33701 |
| UKVDL206 | **MS** | UKVDL206 | WSU003 | WSU003 |
| ATCC 33701 | **MS** | ATCC 33701 | WSU006 | WSU007 |
| ATCC 33701 pc | **MS** | ATCC 33701 pc | UKVDL206 | ATCC 33701 pc |
| WSU006 | **S** | WSU006 | 103+ | WSU005 |
| 103S-GFP | **S** | 103S-GFP | 103S-GFP | WSU006 |

*F: fast-growing, MF: moderate-fast-growing, MS: moderate-slow-growing, S: slow-growing*

**Table S2.** Significant differences observed between strains in liquid growth over time

|  | **103+** | **103S-GFP** | **33701** | **33701 pc** | **UKVDL206** | **WSU001** | **WSU002** | **WSU003** | **WSU004** | **WSU005** | **WSU006** | **WSU007** |
| --- | --- | --- | --- | --- | --- | --- | --- | --- | --- | --- | --- | --- |
| **103+** |  |  | 24*,* 40 | 16 |  | 16*,* 24 | 40 | 16 | 16 |  |  |  |
| **103S-GFP** |  |  |  |  | 12, 40 |  |  | 12 | 12, 16 |  | 24, 40 | 24, 40 |
| **33701** |  |  |  | 16 | 24, 40 | 40 |  | 40 | 16, 40 | 40 | 24, 40 | 24, 40 |
| **33701 pc** |  |  |  |  | 16 |  |  |  | 16 |  | 12 | 12, 16 |
| **UKVDL206** |  |  |  |  |  | 16, 24 | 8, 40 |  | 16 | 24 | 12 | 8, 12 |
| **WSU001** |  |  |  |  |  |  |  |  | 16 |  | 12, 24 | 12, 24 |
| **WSU002** |  |  |  |  |  |  |  | 12 | 16 |  | 40 | 40 |
| **WSU003** |  |  |  |  |  |  |  |  | 16 |  | 12, 40 | 12, 40 |
| **WSU004** |  |  |  |  |  |  |  |  |  | 16 | 12, 16, 24, 40 | 12, 16, 24 |
| **WSU005** |  |  |  |  |  |  |  |  |  |  | 12, 24 | 12, 24 |
| **WSU006** |  |  |  |  |  |  |  |  |  |  |  |  |
| **WSU007** |  |  |  |  |  |  |  |  |  |  |  |  |

*Numbers signify time point at which a significant difference was observed, P<0.05
Underlined* *indicates the isolate in the top row possessed the larger value at the specified time point*

**
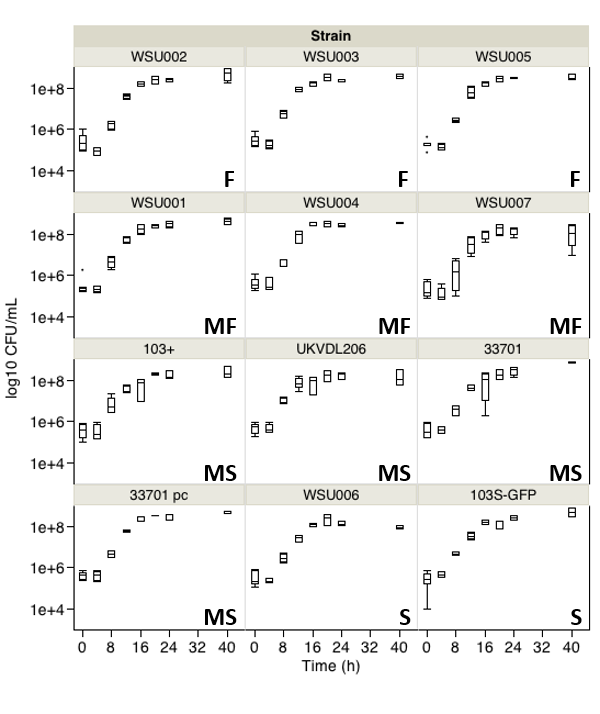
**

**Figure S1.** Growth profiles of *R. equi* strains over time grown in BHI broth at 37ºC with 100 rpm shaking. (F: Fast-growing; MF: Moderate-fast; MS: Moderate-slow; S: Slow. Error bars represent SEM.)


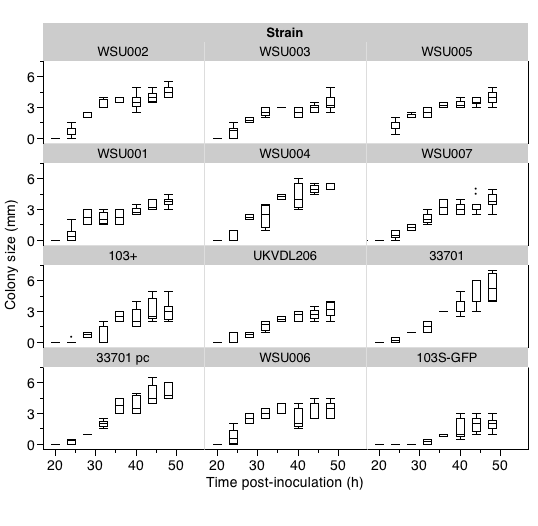


**Figure S2.** Discrete *R. equi* colony sizes (mm) for 12 strains over time grown on BHI agar plates incubated 37ºC for 48 h. **(**Error bars represent SEM.)
